# Gene dosage compensation: Origins, criteria to identify compensated genes, and mechanisms including sensor loops as an emerging systems‐level property in cancer

**DOI:** 10.1002/cam4.6719

**Published:** 2023-11-21

**Authors:** Diana M. Bravo‐Estupiñan, Karol Aguilar‐Guerrero, Steve Quirós, Man‐Sai Acón, Christian Marín‐Müller, Miguel Ibáñez‐Hernández, Rodrigo A. Mora‐Rodríguez

**Affiliations:** ^1^ CICICA, Centro de Investigación en Cirugía y Cáncer Research Center on Surgery and Cancer Universidad de Costa Rica San José Costa Rica; ^2^ Programa de Doctorado en Ciencias, Sistema de Estudios de Posgrado (SEP) Universidad de Costa Rica San José Costa Rica; ^3^ Laboratorio de Terapia Génica, Departamento de Bioquímica Escuela Nacional de Ciencias Biológicas del Instituto Politécnico Nacional Ciudad de México Mexico; ^4^ Speratum Biopharma, Inc. Centro Nacional de Innovación Biotecnológica Nacional (CENIBiot) San José Costa Rica; ^5^ Maestría académica en Microbiología, Programa de Posgrado en Microbiología, Parasitología, Química Clínica e Inmunología Universidad de Costa Rica San José Costa Rica; ^6^ Laboratorio de Quimiosensibilidad tumoral (LQT), Centro de Investigación en enfermedades Tropicales (CIET), Facultad de Microbiología Universidad de Costa Rica San José Costa Rica

**Keywords:** aneuploidy, cancer, gene dosage compensation, miRNAs, systems biology

## Abstract

The gene dosage compensation hypothesis presents a mechanism through which the expression of certain genes is modulated to compensate for differences in the dose of genes when additional chromosomes are present. It is one of the means through which cancer cells actively cope with the potential damaging effects of aneuploidy, a hallmark of most cancers. Dosage compensation arises through several processes, including downregulation or overexpression of specific genes and the relocation of dosage‐sensitive genes. In cancer, a majority of compensated genes are generally thought to be regulated at the translational or post‐translational level, and include the basic components of a compensation loop, including sensors of gene dosage and modulators of gene expression. Post‐translational regulation is mostly undertaken by a general degradation or aggregation of remaining protein subunits of macromolecular complexes. An increasingly important role has also been observed for transcriptional level regulation. This article reviews the process of targeted gene dosage compensation in cancer and other biological conditions, along with the mechanisms by which cells regulate specific genes to restore cellular homeostasis. These mechanisms represent potential targets for the inhibition of dosage compensation of specific genes in aneuploid cancers. This article critically examines the process of targeted gene dosage compensation in cancer and other biological contexts, alongside the criteria for identifying genes subject to dosage compensation and the intricate mechanisms by which cells orchestrate the regulation of specific genes to reinstate cellular homeostasis. Ultimately, our aim is to gain a comprehensive understanding of the intricate nature of a systems‐level property. This property hinges upon the kinetic parameters of regulatory motifs, which we have termed “gene dosage sensor loops.” These loops have the potential to operate at both the transcriptional and translational levels, thus emerging as promising candidates for the inhibition of dosage compensation in specific genes. Additionally, they represent novel and highly specific therapeutic targets in the context of aneuploid cancer.

## 1 INTRODUCTION

Cancer development is a highly complex and multifactorial process that involves a series of genetic and genomic alterations. Genetic instability plays a fundamental role in this process, as it promotes the accumulation of mutations in cancer cells. In this context, aneuploidy, which refers to the abnormal gain or loss of chromosomes, is a common feature in many types of cancer. Aneuploidy can lead to imbalances in gene dosage, affecting the expression and function of multiple genes.^1^

One of the most important features in emerging neoplastic events is the general genomic instability caused by the complete or partial gain or loss of chromosomes, and hence their conversion into aneuploid cells.^2^, ^3^ Aneuploidy represents a high cost for normal cells because it reduces their ability to normally replicate and survive. These alterations lead to so much instability that all monosomies associated with chromosomal loss are known to be lethal. In addition, 20 out of 23 cases of trisomy are fatal, and only trisomy 21 (Down syndrome) is survived to adulthood. Thus, disparities in at least one chromosome—or a section of it—will lead to the imbalance of hundreds of proteins, and subsequent energetic disruption through changes in the metabolic requirements and loss of homeostasis within the cell—and as a final consequence, cell death.^4^

The specific consequences of aneuploidy largely depend on the genetic background of the cells.^5^ Within individual cancer types, the frequency of aneuploid cell content can range from 90% in solid tumors to 75% in hematologic tumors.^6^ Aneuploidy has been found to occur in the early stages of neoplasia: Haploid gain and loss are usually found in polyps, adenomas, and other precancerous injuries.^7^ Nonetheless, sequencing studies also indicate that some chromosomal changes tend to occur late in the course of the disease, such as the chromosome 7 amplification in lung adenocarcinoma, which is suggested to arise in order to tolerate aneuploidy.^8^

Aneuploidy is primarily a deleterious consequence of shortcomings in cells. Behind this deadly threat are changes in cell proliferation, proteotoxicity, protein imbalances, increases in cellular stress, alterations in the metabolic necessities and the production of secondary metabolites that can cause a higher load of metabolic stress. Together, these effects promote further recombination defects, leading to a snowball effect that increases and accelerates genomic instability.^9^, ^10^, ^11^, ^12^ Chromosomes contain the information of tens to hundreds and even thousands of proteins, some of them, responsible for the control of the expression of others in foreign *loci*. Thus, these compensation changes are not accidental—the addition or loss of chromosomes—partial or entire—will produce a turnabout in the dose of a gene, which could potentially contribute to the process of malignancy in cancer cells.^6^

Some studies have uncovered that aneuploidy is responsible for catalyzing genetic instability upon the transformation of tumor cells in the majority of oncogenic processes, resulting in many unstable patterns and cell death by catastrophic error.^4^ Notwithstanding, in some cases there is a specific combination of alterations enabling the cell to overcome the error thresholds in cancer evolution, resulting in viable malignant cells that will potentially develop drug resistance and associated metastasis.^13^ To ensure survival, certain karyotypic configurations arise, suggesting the presence of stabilizing mechanisms, including specific aneuploidies at different stages of cell transformation,^13^ clonal karyotypes that evolve in cell passages,^14^ and chromosomal balances between destabilizing aneuploidy and stabilizing selection for oncogenic function and ultimately the gain or loss of chromosomes to restore the balance of altered proteins.^4^, ^15^

Genetic instability is both an advantage and a burden. There is still debate and lack of information when it comes to understanding how cancer cells are able to overcome the death threshold in the presence of genetic heterogeneity. Despite the negative effects of aneuploidy, polyploidy could be the answer to understanding how cells with completely disrupted metabolisms can still adapt and survive, even when this “survival mechanism” comes with a high risk of becoming lethal regardless of the cellular state of malignancy, depending on the implicated genes. The gene dosage compensation hypothesis offers a compelling explanation on how polyploid karyotypes are able to regulate abnormal levels of proteins and adjust them to avoid proteotoxicity.^16^

In this report, we will review the concept and evolutionary origins of dosage compensation, and the quantitative criteria to identify dosage‐compensated genes. We will also examine sensor loops as potential circuits enabling dosage compensation for specific genes, and the experimental approaches described to validate them.

## 2 ORIGINS OF THE CONCEPT OF GENE DOSAGE COMPENSATION

**FIGURE 1:**
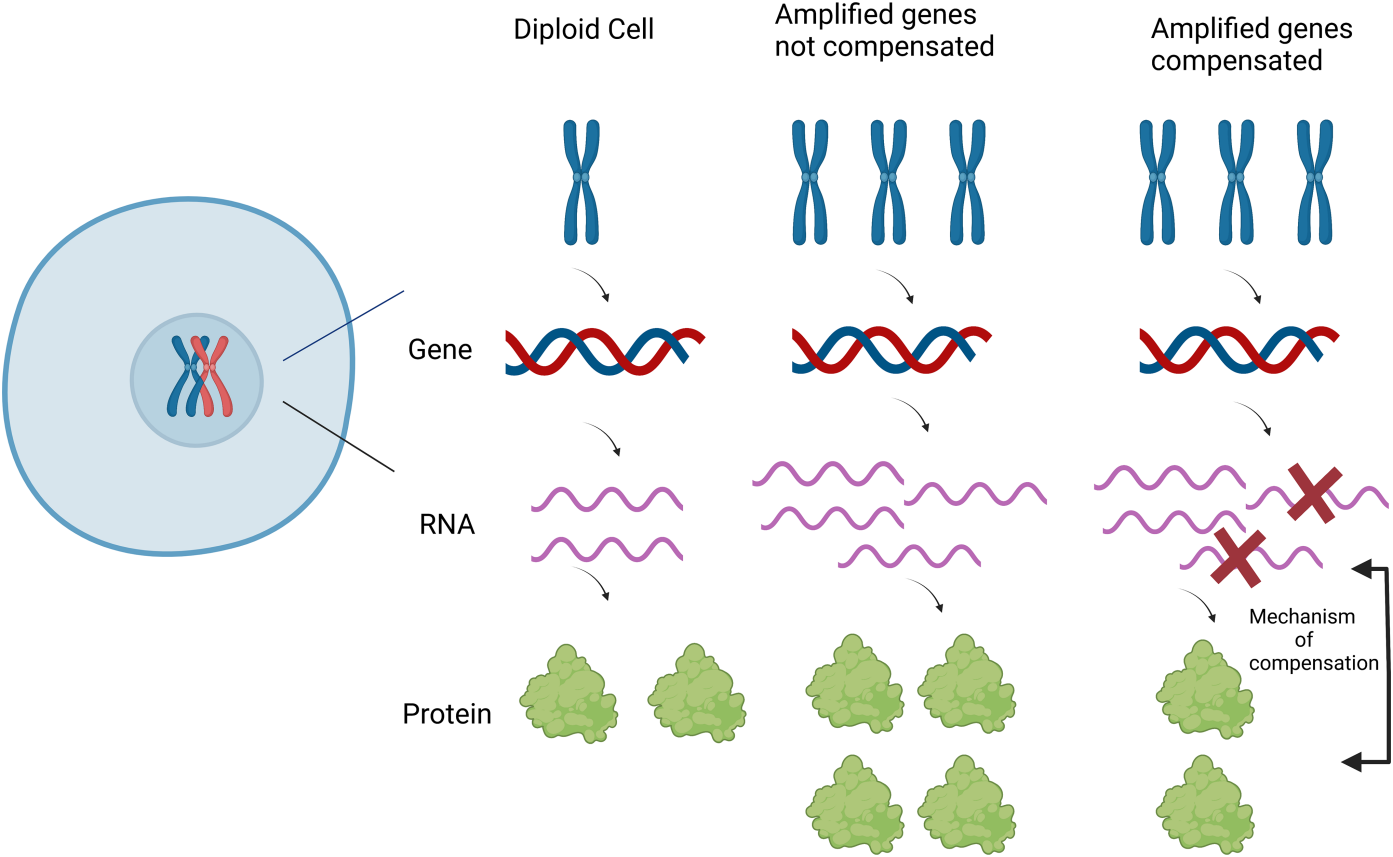
Gene dosage compensation. It represents a compensatory mechanism that might ameliorate the imbalanced expression of certain genes and restore protein homeostasis in aneuploid cells. These cells have compensatory mechanisms to obtain a similar protein expression (and function) compared to diploid conditions despite having an altered number of copies of a genes; compared to a non‐compensated gene where the number of protein or messenger RNA molecules (and thereby its function) significantly increases with an increased gene copy number (figure generated using Biorender.com).

Gene dosage compensation was first described as the way the cells counteract the negative effects of aneuploidy by modulating gene expression upon changes in gene copy number.^17^ The concept of genomic compensation has been known from the early days of the genome as a regulatory mechanism affecting gene expression, quantitative traits, and aneuploid syndromes.^18^ Also, there is a hypothesis that its effects are a consequence of molecular differences between members of macromolecular complexes, interaction systems, and signaling pathways. Therefore, gene dosage compensation has a potential role in improving unbalanced gene expression and restoring protein homeostasis (Figure 1).^19^

The first evidence of gene dosage compensation was reported in *Drosophila* more than 50 years ago when Mukherjee and Beermann observed a negative regulation of genes related to X chromosomes.^20^ Later, a study on the transcription of giant polytene chromosomes of larvae showed that the RNA synthesis rate by single X chromosomes in males was about twice compared to the individual X chromosome in females. Therefore, the male X chromosome is transcription‐equivalent to female with two X chromosomes.^21^ For this reason, a replacement hypothesis is based on the recommended “inverse dosage effect” which works on all chromosomes determined by general transcription regulators.^22^

In males of Drosophila, due to the absence of one X chromosome, there is an increase of expression in the genes contained within it, to levels comparable to females.^23^ New research has elucidated a mechanism through which the male‐specific lethal loci (MSL) sequester regulators away from autosomes in males. This relatively recent observation is particularly convincing that the MSL unused complex in autosomes leads to a local increase in transcription and it eliminates the phenotypes caused by haploinsufficient mutants in the same region.^24^

Sex‐related dosage compensation requires a strict process of regulation, starting with the detection of the number of copies of X chromosomes at the embryonic stage. The sensing and activation of dosage compensation mechanisms in embryos are not exclusive for *D. melanogaster*. There are three sex‐linked mechanisms reported, which occur at the transcriptional level: In mammalian female embryos, the inactivation of one of the doubled X chromosomes occurs^25^; similarly, *Caenorhabditis elegans* hermaphrodites shows a ceased gene expression in both X chromosomes^26^; and finally, *Drosophila* adapts by duplicating the transcription of X genes in males.^21^ The ability to detect the genetic doses of sex‐related genes is pivotal for embryo survival; otherwise, the compensation mechanisms in charge of the conditioning for the X chromosome ratio will fail, leading to death.^27^

Dosage compensation in *Drosophila* must therefore be responsive to the number of X chromosomes in the nucleus. At the genomic level, the process triggered by the X chromosome proportion involves not only the dosage compensation mechanisms, but also sex determination, embryonic sex development, and a later process known as sex‐lethal (Sxl). The *Sxl* gene encodes an RNA‐binding protein responsible for the regulation of splicing and translation of messenger RNAs (mRNAs) which are part of dose compensation pathways in sex chromosomes. Both the SXL protein and its target at the X chromosome create a positive feedback loop in which higher (doubled) copies of this gene will lead to a stronger loop activity, enforcing female development.^28^

Additionally, *Drosophila* females repress MSL2 protein translation through SXL‐mediated repression. MSL2 is part of a protein complex that is key for the upregulation of transcription in males' single X chromosome. By repressing the formation of this complex, females avoid inappropriate dose compensation in the presence of two X chromosomes. In the absence of SXL‐mediated repression, males express MSL2 protein, leading to the assembly of a functional MSL complex.^28^ This chromosomal dosage compensation is a biological mechanism that provides novel information about the basic components of a compensation circuit, including a sensor of gene dosage and a suppressor of gene expression.

## 3 EVOLUTIONARY ORIGINS OF GENE DOSAGE COMPENSATION

It has been determined that sex chromosomes of plants originated from a pair of autosomes.^29^ The hypothesis predicts that no less than two firmly connected sex‐deciding qualities are needed for the introduction of sex chromosomes; the purported “two‐quality model” which comprises of a male sterility transformation (passive in X/Y frameworks, predominant in Z/W frameworks (sex‐assurance framework in birds, females have a couple of different ZW chromosomes, and males have two comparable ZZ chromosomes)) and a female sterility change (prevailing in X/Y frameworks, passive in Z/W frameworks). The recombination between the two deciding *loci* of sex and proto‐sex chromosomes has been proposed to result in a change into genuine sex chromosomes. As a result, the Y chromosome male region would stop recombining and would become a non‐recombinant sex‐specific region (SNR).^30^

Supporting information about plants shows that genes located in the SNR and/or its homologous region X are called sex‐linked genes. The pseudoautosomal region (PAR) surrounding the SNR would continue recombining in both males and females, yet the homologous SNR district in the X would keep on recombining just in *Asparagus officinalis* females.^30^ The process is created similarly for the ZW frameworks, with the main distinction that the area of the female in W does not recombine.

When SNR degeneration occurs, in the ZW and XY systems of the heterogametic sex, lower expression levels tend to be observed compared to the homogametic sex, and it becomes more evident when there is loss of the SNR gene that causes a harmful partial aneuploidy of the heterogametic sex. Even so, in some species there is a “paired” expression level between males and females thanks to an evolved dose compensation mechanism,^31^ with similar expression levels existing between the sex chromosomes and their corresponding autosomal pair.^32^ This suggests that the aneuploidization associated with the origin of the sex chromosomes is linked to a mechanism of genetic compensation.

Liu and collaborators reported that there are seven genes subject to genetic dose compensation at the transcriptional level in *Carica papaya*.^33^ In addition, most X heterozygous genes show little or null expression, suggesting gene silencing may play a role in maintaining transcriptional homeostasis between female and male, while the Y allele in the earlier developmental stage is significantly more expressed relative to the X allele. The decrease in the expression of the Y allele at the oldest developmental stage may be due to the accumulation of deleterious mutations in the regulatory region or the spread of a methylation‐mediated transcription factor.^33^

In species with more derived traits, such as placental mammals, proteomic analysis in sex chromosomes shows a 2‐fold increase in only some proteins that are part of complexes where stoichiometry is essential, when compared against endogenous genes^34^, ^35^; however, the 2‐fold tendency is not observed if all X genes are taken together at the transcription level^36^ nor at the protein level.^37^ These findings propose that only some genes are subject to this kind of dosage compensation.^38^, ^39^

In humans, there are several genetic strategies for cells to cope with X genes; some studies report a downregulation of autosomal proteins, which participate in networks along with sex‐linked genes.^40^ Moreover, there are also findings of some X‐genes being relocated to autosomes.^41^ Notwithstanding, there are substantial mechanisms for dosage compensation, not only in humans but along the whole evolutionary path of sex‐related chromosomes, which show the many strategies to overcome potential deleterious effects. As an example, *C. elegans* eliminates insufficient haploid genes from the X chromosomes,^42^ suggesting that the strategy to transfer a dose‐sensitive gene to an autosomal dominant gene might be widely used among species.

Taken together, the data above indicate that sex chromosomes represent an evolutionary example of the necessities of adaptation to face imbalances once the differences in sex genotypes and phenotypes arrived in the phylogenetic path, hence forcing the cell to implement mechanisms to overcome potential deadly proteic disparities and learn how to regulate chromosomal alterations, including specific gene downregulation/overexpression and the relocation of dosage‐sensitive genes.

## 4 LEVELS OF GENE DOSAGE COMPENSATION

Genetics have a set of natural checkpoints in which strict controls are applied in order to sense, modulate, and respond to cell needs. Although there is lack of information when it comes to determining the level at which the compensation will occur, recent proteogenomic studies have found evidence that cellular function might be an indicator of either RNA or protein regulation to compensate for changes in gene copy numbers.^43^ Therefore, we can postulate that a similar type of checkpoints are used by sensors and regulators of the different mechanisms of gene dosage compensation, occurring at multiple levels of regulation: transcriptional, translational, post‐translational, and epigenetic.

It is difficult to identify the molecular effects of somatic copy number alterations (SCNAs), although various SCNAs are known to change gene expression levels, it is not clear whether each individual SCNA affects gene expression. A study of 77,840 expression profiles was conducted and found that residual expression levels (in the “functional genomic mRNA” profile) were strongly correlated with copy number. A 99% correlation was found between DNA copy number and expression levels of all abundantly expressed human genes, indicating a good overall sensitivity to gene dose. When applied that same analysis to 16,172 patient‐derived tumor samples, they identified recurrently altered genes in genomically unstable cancers. This observation suggests that modifications in gene expression may be a crucial mechanism through which genetic variants exert their influence on the final phenotype. However, the magnitude of the impact of SCNAs on gene expression levels has not yet been determined with certainty. Even so, a large number of genetic variants related to cancer, as well as other complex diseases and characteristics, have been identified. It is clear that disease‐associated SNPs (single nucleotide polymorphisms) and SCNAs (somatic copy number alterations), which are one of the hallmarks of cancer, have a significant impact on gene expression levels.^44^

While multiple questions remain, studies on gene compensation have started to reveal how cells can detect and regulate the levels of proteins in both healthy and pathological conditions. In silico analyses have become great tools to put together information and acknowledge undetected patterns of what determines gene compensation. Cheng and collaborators used the Clinical Proteomic Tumor Analysis Consortium (CPTAC) database which include information of seven different type of tumors including: colon, breast, ovarian, head and neck, uterine, and lung cancer samples with information of the whole genome, whole exome, and protein levels.^43^ Among the results given, the authors have identified gene function as a strong predictor of compensation mechanisms. For instance, genes involved in translation processes and RNA processing are more susceptible to regulation at protein level, and in the other hand, genes related to structural and cellular adhesion have a stronger RNA regulation; suggesting genes that evolved with similarities in function might have also co‐evolved with the same type of compensation strategies.^43^

Additionally, it was found that for every gene evaluated either it showed protein or RNA regulation (i.e., genes with compensation at protein level presented very low or null RNA compensation and the same for RNA‐compensated genes showing no protein regulation). Cheng and collaborators explained this with the co‐evolution hypothesis, where final protein destination might be a strong indicator of the regulation gene pathway; for example, once cell adhesion proteins—including the ones working in complexes—are transported, protein regulation may be a difficult target, leaving RNA compensation as the most viable regulation pathway. On the contrary, cytoplasmic proteins might have shorter RNA half‐life facilitating compensation at the protein level.^43^

In the genomic context, chromosomal gain aneuploidy is a common phenomenon. However, when referring to aneuploidy caused by chromosomal loss, it does not occur with the same frequency. One of the most well‐supported hypotheses as to the lower frequency of monosomies is the detrimental intrinsic nature of the consequences of haploinsufficiency. Some studies have shown that chromosomal loss presents a phenotype where the proliferation is compromised. The loss of essential genome guardians such as the protein P53 and other tumor suppressors as well as stress due to high production of proteins and other consequences of genomic instabilities lead to more aggressive types of cancer.^45^ Transcriptional and proteomic analysis has been performed in order to understand how cancer cells cope with monosomies. Using monosomic cell lines named RPE1‐derived monosomy, investigators used whole‐genome sequencing to identify the chromosomal losses, Tandem Mass Tag (TMT) to analyze changes in proteome and RNA‐seq to elucidate the amount of RNA encoded. With this data, it was possible to observe that monosomies counteract their genomic deficiencies by upregulation of gene expression. The data indicate that 30% of the genes are adjusted at the transcriptional level and 45% at a post‐transcriptional level.^45^

## 5 CRITERIA TO IDENTIFY GENES UNDER DOSAGE COMPENSATION

Defining gene dosage compensation merely as a situation in which gene expression does not scale with gene copy number could be overly simplistic. The questions of whether non‐compensated genes scale with a 1:1 ratio between gene expression and copy number, and of how much the expression of a gene scale with copy number to be still considered compensated, have represented a big challenge for the scientific community interested in the topic, especially considering that these thresholds could be functional and vary from gene to gene depending on many confounding factors including cell microenvironment. The cell requires a delicate balance between sensing and responding to stimuli, and the sensory process is intimately linked to cell physiology. When a certain pathway is activated, specialized proteins travel to the nucleus and turn the genetic machinery on or off. At this point, the availability of their target (the chromosome/gene sequence itself) will predict the levels of transcription, and translation products, and the final amount of protein produced. As a result, the correlation between having an extra chromosome and the consequential increase of gene expression has been widely explored.^46^, ^47^, ^48^, ^49^, ^50^, ^51^, ^52^, ^53^, ^54^, ^55^, ^56^, ^57^, ^58^

Nonetheless, cells have shown incredible plasticity in adapting and overcoming stress and potential death threats—especially cells with higher levels of malignancy. Among different studies carried out in fruit flies, mice, human cancer cell lines, and yeast, it was revealed that certain genes found in aneuploid chromosomes have either a normal expression rate or underexpression compared to haploid cells.^59^, ^60^, ^61^, ^62^, ^63^, ^64^ Indeed, previous investigations have shown varying degrees of gene dosage compensation, with several studies determining that compensation is occurring only in certain genes.

The discrepancy between different human cancer cell studies has caused controversy over which genes are dosage‐compensated, and therefore, other studies have tried to establish criteria to identify dosage‐compensated genes. However, aneuploidy by its own nature is catastrophic, meaning that it would present differences not only in how the allelic imbalance is going to affect the gene, but many studies have been able to put together how tumor line, type of cancer, its frequency, the mutational burden, and global CNA are affected.^65^, ^66^ Given the allelic changes in within and between human cancers, it is possible to propose models describing how much variation exists among mutants and healthy counterparts; however, this type of estimation presents major challenges: Predicting mutations in this situation requires a very precise reasoning of allele and specific global copy, in single copy change resolution,^67^ and quantitative mutant allele frequencies, both under the control of tumor nature and its heterogeneity.^68^

For example, reports of human chromosome 5 insertions indicated that most proteins are overexpressed compared to diploid cells, suggesting that there is no effective general gene dosage compensatory mechanism in this system. However, some specific proteins remain within the diploid expression range, especially those corresponding to the kinases, protein subunits, and ribosomes. A large number of genes are compensated at the protein level, even so, others receive their compensation at the mRNA level.^53^ The strategy of inserting additional chromosomes to study gene dosage compensation is ideal because the authors worked on the same genetic background, in contrast to other studies comparing different cell lines. To define a gene as dosage‐compensated, they used a criteria based on the comparison between the log2 ratio of gene expression (both at RNA and protein levels) to the log2 ratio of the gene copy number. If the RNA or protein expression is closer to 0 (similar to diploid) than to 1 a gene could be considered compensated but there is no clear cutoff and as stated above, this cutoff could depend on the function of each individual gene.

In contrast, experimental models in yeast have determined that the phenomenon of dosage compensation appears to be quite extensive, and will affect a significant portion of the aneuploid genome content. A report by Hose et al. found that, compared to homozygous or closely related euploid strains, 10%–30% of gene amplifications in aneuploid wild‐type yeast strains were compensated by gene dose, and aneuploidy did not cause developmental defects. The authors also speculated that dosage compensation occurs in genes that are more toxic upon overexpression, and their expression may be more restricted during development.^69^ To define which genes are dose‐compensated against genetic polymorphisms, Hose set al. proposed a criterion based on isogenic lineage analysis of yeast lines YPS1009 and the West African line NCYC110, in which the isogenic diploids carry two, three, or four copies of Chr 12 or Chr 8, respectively, and they performed mRNA and DNA measurements. Each panel included paired euploid references, and a mixed linear regression (MLR) model was developed to classify genes based on gradients and transitions between mRNA gene repeat levels. Class 1 genes show relatively increased mRNA abundance with increased gene transcription in the stress panel, with a slope of 1.0 and a log2 cross of 0 showing similar expression in the two euploid lines.^69^ Therefore, these yeast genes do not show evidence of dose compensation or heritable altered expression. Class 2 genes also show a linear relationship between mRNA and DNA copy (slope of 1.0) but have an altered intersection that reflects a constitutively reduced (Class 2a) or constitutively high (Class 2b) mRNA for each gene copy. Thus, class 2 genes showed genetically variable expression, but no evidence of dose compensation. In contrast, class 3 showed a disproportionate relationship between mRNA abundance and gene copy number. For class 3a genes, mRNA did not increase proportionally to the increase in gene transcription in the stress panel, as indicated by the decreasing gradient of a linear fit. Similarly, class 3b genes have a gradient of >1, indicating that abundant mRNAs are more amplified than expected with increased gene transcription. As the strains in each panel are genes in common except for heterozygotes, low slopes of class 3a genes indicate dose compensation, while strong gradients of class 3b genes represent dose amplification.^69^

Hose et al. also implemented a criteria based on a CNV‐buffering capacity (Bv) for the comparison of aneuploid yeast strains, as the dosage‐compensated genes had higher variation in copy number but a constrained expression level. Bv for each gene is the phylogeny‐weighted sum of gene copy number across strains divided by *V*_*g*_/*V*_*m*_ measured for that gene, where *V*_*g*_/*V*_*m*_ is a comparison between the variance in gene expression seen in natural isolates subject to mutation and selection (the genetic variance, *V*_*g*_) to the mutational variance (*V*_*m*_) of diploid mutation‐accumulation (MA) lines propagated in the near‐absence of selection. However, as they also mentioned, they are comparing yeast strains of diverse genetic backgrounds and this could introduce noise to gene expression levels. In addition, there is again no clear threshold on the CNV‐buffering capacity in order to determine when a gene is clearly compensated or not.

Shao and collaborators tested over one thousand cell lines and nearly 10^4^ tumor samples, and determined that in a non‐perturbed state of the cell there will be a correlation between gene copies and the protein products. What highlights these studies is that many oncogenes (e.g., *Myc, Akt1, Cdk9, Kras*, and *Mdm2*) and tumor suppressor genes (e.g., *Cdkn2a, Rb1, Pten*, and *Tp53*) are involved in CNAs.^70^ They implemented a strategy based on a Pearson correlation between the rank values of the *z*‐score of gene expression and copy number, to assess how well the relationship between two variables can be described using a monotonic function. Most genes presented a high degree of fitting with r intensively ranging from 0.8 to 1, except for some oncogenes including Myc, tumor suppressors including Tp53, and genes involved in retinol metabolism, olfactory transduction, calcium signaling pathway, and neuroactive ligand–receptor interaction. Once again, this study is based on the comparison across cell lines of different genetic backgrounds and does not establish a clear cutoff of compensation.

In our previous work,^71^ we implemented a similar approach to CNV‐buffering capacity of Hose et al. to identify dosage‐compensated genes. We began with the NCI60 human cancer cell lines given the amount of information available for evaluation and comparison, in which investigators worked with gene copy numbers, genic expression, and proteomics. First, we computed the average RNA or protein expression across the cell lines with diploid copy number for each gene and used these values to normalize all the gene expression data and transformed it using log2 to compare across CNV, RNA, and protein. Afterward, we applied a very simple criteria calculating the standard deviation for the 3 data sets and plotting these data we could notice a cluster of genes naturally emerging from the data that was easily separated by a gaussian mixture model. This cluster presents a high variation in gene copy number but low tolerance to variation in gene expression, for both RNA and protein. We could narrow it down to 19 candidates, including genes important for cancer development, particularly seven highly associated transcription factors, some of which are localized at common chromosomal loci. The genes sensitive to gene dosage compensation are *Myc, Znf217, Mtss1, Sema3d, Trim37, Stat3, Kcnh4, Rab5c, Focx1, Birc2, Cul5, Atm, Npat, Mmp12, Dcun1d5, Pgr, Pdcd10*, and *Atp1b2*. It should be noted that all the genes mentioned above have some dose‐regulating genes at the RNA level and only some at the protein level.^71^ Our approach has the advantage of defining a clear threshold to identify gene dosage compensation. Although we could confirm these findings with the CCLE data set and extend our list of candidates, our approach has the same limitations as others as it compares cell lines with different genetic backgrounds. Although we tried to deal with that by identifying genes with low variation in gene expression, the small list of candidate genes indicates that our approach has probably high specificity but low sensitivity. Indeed, we could be missing compensated genes presenting variation due to other confounding factors independent of copy number alterations.

Mohanty and collaborators also looked at the uncoupling of mRNA expression from copy number (UECN) as a strategy for cancer cells to tolerate a high degree of aneuploidy using the dataset from The Cancer Genome Atlas (TCGA), encompassing 5000 individual tumors. They implemented a double criteria based on partial correlation between copy number and RNA expression while controlling for tumor purity and the regression of the expression data against copy numbers while controlling for tumor purity and the top 20 expression principle components, where the strength of association is defined by the Spearman correlation coefficient and T‐value corresponding to the copy number term in the regression model. They used only genes that were consistently expressed in each tumor (90th percentile expression >30 normalized counts). The density distributions of both terms have a bimodal distribution in multiple cancers suggesting that while the expression of numerous genes is coupled with their copy numbers, the expression of a considerable proportion of genes is uncoupled from their copy numbers, highly enriched for pathways associated with immune response across cancers. Using this approach and machine learning to model complex regulatory networks, the authors identify 21 transcription factors as potential therapeutic targets to perturb gene dosage compensation in aneuploid tumors.^72^ Their approach has the advantage of finding bimodal distributions to identify potential uncoupled genes and also of correcting for tumor purity and other confounding factors such as the presence of genes with low or no expression in the tumor's corresponding normal tissue and genes whose expression changes with differentiation status. Unfortunately, in some cancers, such as colon adenocarcinoma and ovarian cancer, the bimodal distribution is not' as striking as observed for other tumors.

TACNA profiling was recently published^73^ as a brilliant strategy to extract the transcriptional effects of copy number alterations for more than 28.000 patient‐derived tumor samples. Their algorithm separates the effect of CNA (copy number alteration) from other factors influencing the net measured expression level. Basically, it denoises the data from other factors and leaves only the fraction of gene expression variation accounted for by the changes in copy number based on neuronal networks. The analysis divides gene expression profiles into sources of “community consensus estimates” (CES), creating each CES statistically independent from one another. The hypothesis established each CES could describe the effects of potential transcriptional regulators on gene expression levels, therefore representing precisely each of those confounding factors such as trans‐regulation and tumor heterogeneity. In each CES, each gene has a weight, which describes how its expression is affected by the transcription of a potential regulator, the direction of activity and the effects of copy number change.^73^ They used this approach to observe that most of the oncogenes have a high degree of transcriptional adaptation to CNA, indicating that increasing the expression levels of several oncogenes is only beneficial for the development of tumors to some extent.^74^

More recently, Schukken and Sheltzer^75^ have performed a detailed characterization of gene dosage compensation both at the transcriptional and protein levels of 367 human cancer cell lines of CCLE with matched DNA copy number, RNA expression, and protein expression. Integrating several datasets, they classified all genes as anti‐scaling, buffered or scaling both at the RNA and protein level. They found that upon chromosome gains and losses around 35% of genes were compensated at the RNA level whereas 59% of the genes showed buffering at the protein level. These very interesting findings indicate that our previous criteria to identify dosage‐compensated genes are rather limited to those with high variation coefficients across cancer cell lines, but gene dosage compensation is a much broader phenomenon in aneuploid cancer.

Schukken and Sheltzer^75^ also found that upon the gain of one additional chromosome, the mean RNA level increases an average of 22% (instead of expected 50%) whereas protein expression increases by only 12%. This means that additional gene copies are not directly related to a 1:1 linear increase in RNA or protein expression for most genes either because the system is not efficient in expressing these additional copies or due to the presence of transcriptional and, especially, post‐transcriptional dosage compensation mechanisms that can buffer the effects of aneuploidy on gene expression. As a consequence of this nonlinear behavior, it is a challenge to identify a threshold to define which amplified gene copies are actually compensated. For instance, Schukken and Sheltzer^75^ used a recent dataset in which each chromosome arm in a cell line was classified as “neutral,” “lost,” or “gained” relative to the basal ploidy.^76^ Thereby they compared the mean expression levels between those different groups and reported gene dosage compensation for many genes in gained arms having a similar mean in RNA or protein expression compared to the mean expression of their corresponding basal ploidies. Apart from the non‐normal distributions of gene expressions, this approach does not distinguish between different levels of amplification. In addition, there is not a clear threshold to identify a gene copy as compensated or not, especially due to the nonlinear increase in overall gene expression upon CNVs.

Cheng et al. performed a proteogenomic analysis of tumors and untransformed cells containing somatic copy number alterations (SCNAs). They found that protein complex genes have a strong protein‐level regulation while non‐complex genes have a strong RNA‐level regulation, with some exceptions due to protein localization and specific pathways. They implemented criteria based on the DNA–RNA correlation to analyze RNA‐level regulation and RNA–protein correlation to study protein‐level regulation. To quantify those two levels of compensation, they calculated a compensation score for each gene in each sample defined as the difference between the RNA or protein log2FC with the DNA log2FC.^43^ These criteria have the advantage that they permit statistical evaluation of whether there is a significant compensation in each group of DNA change when their compensation score was significantly larger than zero by a bootstrapping method that randomly samples the score of the genes within each group.

McShane et al. performed global pulse‐chase experiments designed to study gene dosage compensation at the protein level. To model their experimental protein degradation profiles, they trained two Markov chain‐based models previously used to study mRNA decay, where they could distinguish two different states. The first model had only one state describing exponential degradation. The second model of non‐exponential degradation (NED) has an additional state, so that newly synthesized proteins first populate state A from where they can either be degraded or transit to state B, which is characterized by a different decay probability. Using these two models, they could clearly classify each protein degradation profile using the Akaike information criterion (AIC), observing which profile fits a trade‐off between the goodness of fit and model complexity defined by the number of parameters. They observed that 14% of the 3605 proteins fall into the NED category and they found out that 70% of NED proteins are members of heteromeric protein complexes and many of those complexes contain both NED and ED proteins, suggesting that excess protein subunits could be rapidly degraded, accounting for potential gene dosage compensation at the protein level. They also found that indeed NED proteins in complexes appear to be produced in super‐stoichiometric amounts relative to their ED counterparts. Using the trisomic cell line of Stingele et al.,^53^ they also found that NED proteins in trisomic regions displayed significantly increased non‐exponential degradation and this behavior was specific for NED proteins and not observed for ED proteins.^77^ They concluded that decay profiles can help to predict protein level attenuation in aneuploidy and therefore to identify dosage‐compensated genes at the protein level, with the advantage of a clear distinction between NED and ED proteins.

Other qualitative criteria could be combined to contribute to identify dosage‐compensated genes and classify the type of dosage compensation level or mechanisms, as described in the following sections. Many studies consider that the participation of a gene in the formation of protein complexes is a strong criteria for dosage compensation due to accelerated breakdown of non‐assembled subunits,^78^, ^79^ protein degradation by ubiquitin–proteasome system,^78^, ^79^, ^80^, ^81^ the acetylation of the N‐terminal amino acid by N‐acetyltransferases (NAT),^79^, ^81^, ^82^ and the co‐deregulation of protein complex partners.^83^ On the contrary, if a candidate compensated gene does not form part of a protein complex it might be regulated at the RNA level,^43^ specially if the gene is a tumor suppressor or an oncogene,^70^, ^71^, ^72^, ^73^ if it has a long RNA‐life or if it is a membrane protein, a structural protein or it is involved in cell adhesion.^43^ Notwithstanding, there are proteins whose RNA half‐life can be relatively short, thus favoring regulation at protein level. In addition, if the protein participates in processes of translation or RNA processing, it is also potentially regulated at the protein level.^43^

In summary, several criteria have been established to identify dosage‐compensated candidates both at the RNA and protein levels (Table 1). However, as discussed above they all have pros and cons. Many criteria are limited by the noise in gene expression as they compare cell lines of different genetic backgrounds. Some criteria try to deal with that heterogeneity by identifying dosage‐compensated genes with low tolerance to variation in gene expression despite a high variation in copy number, but they are probably very stringent leading to low sensitivity (like our criteria) as they rule out genes, which expression varies due to other confounding factors and they also depend on having enough variation in gene copy number. Other criteria depend on normalization and statistics such as the mean to compare between highly heterogeneous (non‐normally distributed) groups of genes to classify them as compensated or not, and do not take into account the level of amplification. TACNA profiles deal with those confounding factors and heterogeneity by extracting the gene expression component related to copy number changes but it is restricted to transcriptional‐level compensation and as a machine‐learning approach, which may require further experimental validation. Many of these criteria do not define a clear cutoff to define dosage‐compensated genes with some exceptions. The prediction of gene dosage compensation at the protein level by their degradation profiles (NED) offers a clear separation of dosage‐compensated proteins, but not all compensated proteins are NED. In addition, when combined with criteria such as the formation of protein complexes, only 70% of NED proteins form those complexes.

**TABLE 1:** Quantitative and qualitative criteria to identify dosage‐compensated genes and their type of regulation.

Criteria to define compensation	Genes	Potential levels of compensation	References
Similar Log_2_ ratio of gene expression compared to copy number amplification	Subunits of protein complexes and kinases	Protein subunit degradation Kinase activity? Several few genes regulated at the RNA level	[53]
1. Mixed linear regression (MLR) model to classify genes based on gradients and transitions. 2. High CNV‐buffering capacity defined as log CNV‐buffering score	Genes that are most toxic when overexpressed	Gene expression regulation mechanisms	[69]
Markov chain‐based models to classify protein decay in Non‐exponentially degradation of proteins (NED)	70% of NED proteins are protein complex subunits	Proteosome degradation of proteins	[77]
Low ρ: the Pearson correlation between the rank values of the z‐score of gene expression and copy number, to assess how well the relationship between two variables can be described using a monotonic function.	Oncogenes and tumor suppressors, retinol metabolism, olfactory transduction, calcium signaling pathway, neuroactive ligand–receptor interaction	Potential regulation at the RNA level	[70]
High degree of transcriptional adaptation (TACNA profiles)	Many oncogenes	Regulation at the transcriptional level	[73]
Gene clustering with low variation in diploid normalized gene expression data but high variation in copy number	19 candidate genes including 7 transcription factors	Potential regulation at the RNA level	[71]
Partial correlation between copy number and RNA expression while controlling for tumor purity. Bimodal distribution of the strength of association by Spearman correlation and a regression model of expression against copy number	A group of copy number uncoupled genes highly enriched for pathways related to immune response across cancers and 21 transcriptions factors controlling their expression	RNA‐level regulation: suppression by promoter methylation, higher negative regulation by miRNAs, lower positive regulation by transcription factors	[72]
Similar mean in gene expression compared to the mean expression of their basal ploidies	35% of genes compensated at the RNA level 59% of genes compensated at the protein level	RNA and protein level	[75]
Compensation Score: difference between the RNA or protein log2FC with the DNA log2FC. Statistically validated by bootstrapping.	Mostly protein level compensation. RNA‐level compensation in certain tumor types RNA‐level compensation for non‐complex genes	RNA–protein correlation informs protein‐level regulation	[43]
		DNA–RNA correlation informs RNA‐level regulation	[43]
Protein complexes	Genes that code for protein complexes	Accelerated breakdown of non‐assembled subunits	[78, 79]
		Protein degradation by ubiquitin–proteasome system	[78, 80, 81]
		Acetylation of the N‐terminal amino acid by N‐acetyltransferases (NAT)	[79, 81, 82]
		Co‐deregulation of protein complex partners	[83]
Non‐complex protein	Non‐complex genes	RNA‐level regulation	[43]
Oncogenes and tumor suppressor genes	*Myc, Znf217, Mtss1, Sema3d, Trim37, Stat3, Kcnh4, Rab5c, Focx1, Birc2, Cul5, Atm, Npat, Mmp12, Dcun1d5, Pgr, Pdcd10, Atp1b2*	Protein regulation but mainly RNA‐level regulation	[70, 71, 72, 73]
RNA half‐life	Long RNA half‐life	RNA regulation	[43]
	Short RNA half‐life	Protein Regulation	
Protein nature and destination	Translation process and RNA processing	Protein regulation	[43]
	Membrane proteins, structure proteins and cell adhesion	RNA regulation	
Functional dosage compensation: Genetic tug‐of‐war leads to the downregulation of the endogenous gene upon overexpression of an exogenous version	10% of genes of yeast chromosome 1	Protein level regulation, subunits of multiprotein complexes regulated in a stoichiometry‐dependent manner	[84]
	Myc	Transcriptional‐level regulation	[71]
Overexpression leads to cell death	Myc, lethal genes in yeast	Transcriptional regulation	[69, 71]
Compensation region within the n‐dimensional parameter space of mathematical models of miRNA‐mediated compensation circuits	Transcription factors participating in miRNA‐mediated compensation circuits	RNA and protein levels, transcriptional or translational regulation depending on Ago activities.	[71]

Therefore, a precise definition of gene dosage compensation is difficult to achieve, especially because the threshold of the gene expression scaling as a function of copy number could be different for each gene and probably also depends on the cellular context. For example, a “cancer zone” has been defined as a range of MYC (and/or E2F) expression where a slight increase is associated with carcinogenesis but a further increase would lead to cell death, incurring into an error threshold in cancer evolution.^85^ Thus, an optimal criteria to identify dosage‐compensated genes are still missing, but a combination of the criteria described above could strongly contribute to the identification of dosage‐compensated candidates. However, a comprehensive definition of gene dosage compensation involves a functional aspect for the cell, wherein evolutionary constraints ensure that specific expression levels are maintained to support cell viability.

Finally, a full demonstration of a functional gene dosage compensation requires an experimental validation of the compensation mechanism using a genetics tug‐of‐war approach where an exogenous version of the gene is overexpressed and the effect is observed in the expression of the endogenous gene (see section below). A genetic tug‐of‐war was developed to demonstrate functional dosage compensation at the protein level.^84^ Inspired by this, we also developed a tug‐of‐war strategy for dosage compensation at the transcriptional level enabling the experimental demonstration of the presence of compensating circuits in Myc expression.^71^ In addition, we demonstrated that the blockade of those circuits induces cell death, completing thereby a full definition of functional gene dosage compensation. Finally, we also identified in silico the range of kinetic parameters of the compensating circuits that could be used as preliminary criteria to identify other compensated candidates for further experimental validation.

## 6 GENE DOSAGE COMPENSATION MECHANISMS AT THE PROTEIN LEVEL

**FIGURE 2:**
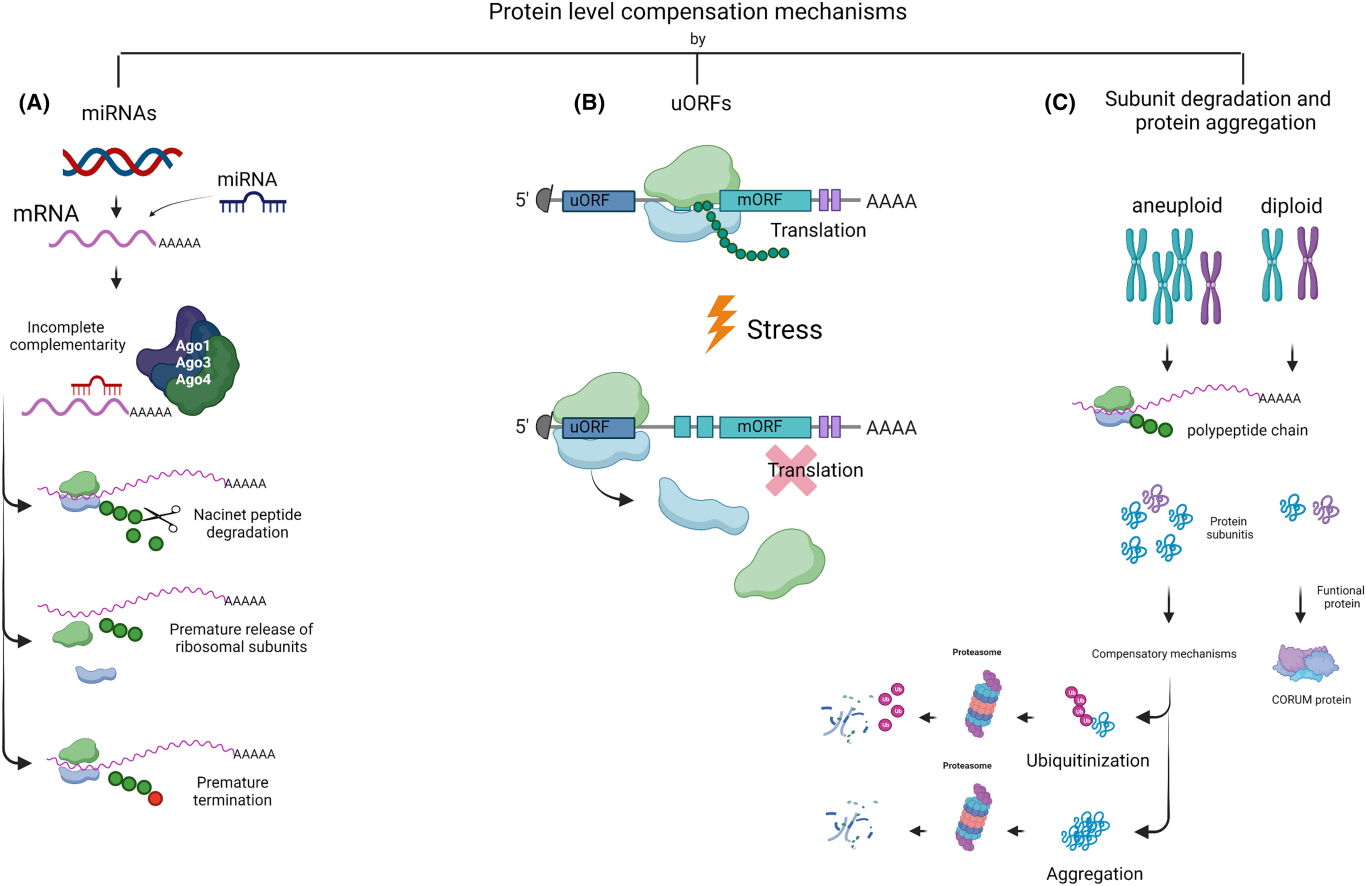
Protein level compensation mechanisms. Several mechanisms have been described that exist for compensation of gene dose at the protein level. (A) miRNAs could regulate gene expression both at the post‐transcriptional level and at the translational level, depending on their complementarity and binding to different argonaute (Ago) proteins. (B) Ribosomes load at the 5′ end of the mRNA, when there is a uORF and in conditions of stress, some genes are downregulated by the ribosome loading in the uORF and either uncoupling, reinitiation the reading of the main open reading frame (mORF) or just elongating the uORF sequence. (C) In aneuploid conditions where protein subunits are encoded in aneuploid chromosomes, there is an overproduction of protein subunits, so this imbalance must be corrected by degradation by the ubiquitin–proteasome system or by aggregation (figure generated using Biorender.com).

After exploring the multiple criteria to identify candidate genes under dosage compensation, we will review in more detail the different reported mechanisms of gene dosage compensation both at the protein and RNA level. Although regulation at transcriptional level is supposed to be the optimal alternative when it comes to energy management, it might be clear how evolution, function, and protein destination are pivotal in protein regulation.^43^ Indeed, gene expression changes are largely explained by gene dosage compensation at the protein level. Proteins are characterized for their versatility as they either work as a part of a complex (homogeneous or heterogeneous complexes) or could be functional as a unit. The following studies involve mechanisms at the translational level and at the protein degradation level, which are summarized in Figure 2.

### 6.1 Protein degradation

Some investigations have found that cancer cells showing aneuploidy have mRNA levels that often correlate with the increase in DNA copy numbers (gene dose), but these changes are not reflected at protein levels.^53^ For instance, a research carried out by Brennan and collaborators, demonstrated that gene dosage compensation occurs in cancer cells mainly at the protein level, whereby protein aggregation mediates the stoichiometry of protein complexes in aneuploid cells. Excess subunits are degraded or aggregated, with protein aggregation being almost as effective as protein degradation in reducing the level of functional proteins.^86^ As shown above, it has been described that gene dose compensation at protein levels generally occurs in genes that code for protein complexes^43^, ^53^, ^84^ and it is subject to changes depending on the availability of protein subunits associated with the protein complex.^78^

Other studies using the pulse‐tracking metabolic labeling technique and quantitative mass spectrometry have revealed that a considerable percentage (>10%) of proteins exhibit non‐exponential degradation. This is because newly synthesized proteins are less stable and become more stable as time passes since their synthesis. A large number of non‐exponentially degraded (NED) proteins are subunit complexes that are produced in stoichiometric amounts compared to their exponentially degraded (ED) counterparts. Within these complexes, the NED proteins present more extensive interaction interfaces and assemble earlier than the ED subunits. As a result, the amplification of genes encoding NED proteins leads to an increase in their initial degradation. Consistently, decay profiles can predict the decline in protein levels in aneuploid cells. Taken together, these data demonstrate that non‐exponential degradation is a common and conserved phenomenon, with important implications for complex formation and regulation of protein abundance.^77^

Another dosage compensation mechanism involves the accelerated breakdown of non‐assembled subunits through the ubiquitin–proteasome system.^78^, ^79^ Control of stoichiometry is carried out by the E3 ubiquitin.^80^ Likewise, a mechanism of selective degradation of protein subunits has been described through the mechanism of acetylation of the N‐terminal amino acid by N‐acetyltransferases (NAT). N‐acetylation has been shown to act as a degradation signal that is specifically recognized by E3 ubiquitin ligases.^79^, ^81^, ^82^

Senger et al. also found a large number of expression changes happening on non‐aneuploid chromosomes different from those aneuploid ones in cancer cells. They associated those changes with co‐complex protein members in both types of chromosomes, and this tight regulation of co‐abundance is performed for aggregation‐prone aneuploid proteins and others involved in complexes. They demonstrated that those compensatory and maintenance mechanisms are regulated at the post‐translational level and the extent of success of a tumor to deal with aneuploidy depends on the activation of protein degradation processes.^83^

### 6.2 Translational regulation of protein expression

Bioinformatic analyses carried out in cancer cells conclude that a vast majority of proteins are subject to some type of regulation to counteract the partial or entire gain or loss of chromosomes. Not surprisingly, it has also been observed how gene dose compensation contributes to enhance the functions of oncogenes and decrease the expression of tumor suppressor associated genes; nonetheless, the compensation process may not be perfect, since the degree of aneuploidy could vary from one cancer type to another. Notwithstanding, certain genes such as *Her2*, *Mtor*, and *Myc* require strict maintenance of their protein levels to help through tumoral development. Therefore, protein complexes play a really important role—but is not the only way—to compensate for a gene at the protein level.^75^

Deleterious effects have been observed due to the stoichiometric imbalance of the protein subunits involved in cell growth.^87^, ^88^ Recent studies from the analysis of ribosomal protein profiles show that there is a close balance between the stoichiometry of the subunits of multiprotein complexes versus the proportional synthesis of protein subunits, the latter being mainly regulated at the level of translation.^89^, ^90^

Another mechanism related to translational regulation for the dosage compensation of gene losses is related to definite sections called “upstream open reading frames” (uORFs). These regulatory components might act as inhibitors^91^, ^92^ located specifically in the 5′UTR segments of mRNAs, found in at least 50% of all vertebrates.^93^, ^94^ For instance, upon stress conditions the phosphorylation of eIF2α competes as an inhibitor against eIF2B, the latter being a eukaryotic translational factor, and as a consequence there is a decrease in translation reinitiation rates.^95^ At the same time, the gene *Gcn4* is regularly repressed with its 4 uORFs restricted but once stress signals appear, the only uORF that is efficiently translated is the first one. However, thanks to eIF2α phosphorylation, the remaining uORFs are not correctly translated and reinitiation only occurs at the main ORF, increasing GCN4 production.^95^ These findings might indicate that genetic mutations could trigger cellular stress pathways, allowing for uORF skipping and increased translation of compensating genes.^96^

Indeed, Schukken & Sheltzer found that only 31% of buffered proteins were members of one or more protein complexes.^75^ McShane et al. found that only 70% of NED proteins participate in the formation of protein complexes.^77^

Therefore, we can conclude at this point that other mechanisms either at the transcriptional or post‐transcriptional level must be at work to account for gene dosage compensation at the protein level in many cases. Now is becoming more evident that protein degradation coexists with regulation at the protein synthesis level, and that at least for certain complexes, the vast majority of the protein‐level regulation occurs at the protein synthesis level with fine‐tuning happening through protein degradation.^90^, ^97^, ^98^, ^99^

## 7 GENE DOSAGE COMPENSATION MECHANISMS AT THE RNA LEVEL

Despite the fact that gene dosage compensation is not exclusive to cancer cells, aneuploidy is a driving hallmark of cancers. The high level of aneuploidy in cancer is likely related with a high level of dosage compensation with the confounding contribution of different levels of gene regulation. Therefore, the study of the regulatory levels of dosage compensation must be performed very carefully in cancer datasets. For example, pan‐analysis of tumoral samples at the bioinformatic level shows a strong preference for protein regulation for compensation in aneuploid genes; in contrast, examining the data sets for specific cancers demonstrates that some types of cells present a preference for either protein regulation or RNA regulation. Colon adenocarcinoma samples exhibit a higher inclination for compensation at RNA levels and although ovarian cancer genes presented increased protein regulation, their genes coding for noncomplex proteins have a preference for RNA‐level regulation.^43^

Colon, breast, ovarian, and renal cancers revealed that the RNA levels vary in a non‐correlated way with the gene copies, while only lung cancers exhibit compensation at protein levels. In addition, deeper analysis also showed differences in the regulation of genes coding for protein complexes and individual ones, revealing stronger protein regulation for complexes but higher levels of RNA‐targeted regulation for the coding‐genes of functional protein units.^43^

A characterization of the transcriptome and proteome in aneuploid human cancers showed that 47%–63% of the genes located in aneuploid chromosomes evidence changes in expression at the transcriptome level, in contrast to only 24%–33% at the proteome level.^83^ However, the degree of expression changes induced by aneuploidy varies depending on multiple factors, among them the cellular environment.^16^ For instance, Senger et al. found that genes on other chromosomes show a surprising degree of differential expression. When performing the comparison of the transcriptome and proteome data, it was observed that the proteomic changes of the aneuploid chromosomes could be explained mainly by the differential expression of their corresponding genes in the transcriptome, even so, these observations are not concordant for the proteomic changes of non‐aneuploid chromosomes.^83^

Moreover, Gonçalves et al. performed an analysis of transcriptomic and proteomic datasets of 282 breast, ovarian, and colorectal tumor samples available in the CPTAC and TCGA consortia, finding that CNVs are buffered by post‐transcriptional regulation at the protein level only in 23%–33% of the proteins that are part of protein complexes, indicating that other mechanisms should be at work.^100^

The studies described above indicate the existence of different contributions of the regulatory levels of gene dosage compensation, where the RNA‐level regulation predominates in genes within aneuploid chromosomes, while for other chromosomes translational or post‐translational control has a comparatively greater importance.^83^ We detail below though mechanisms of transcriptional or post‐transcriptional control at the RNA level that have been proposed to play a role in gene dosage compensation.

### 7.1 Transcriptional control of gene expression

Although genetic compensation has been found along epigenetic‐transcriptional‐translation‐protein levels, it has been suggested that transcriptional compensation may help the energetic cell balance, avoiding the “waste” of energy on further protein translation, degradation, and even complex‐docking.^96^ One important criterion to consider is that not all genes are subject to compensation, even if they are located within the aneuploid chromosome. In addition, we must consider that compensation‐mediated up‐or‐downregulation of a transcription factor could result in altered gene expression in other chromosomes (for review, see^16^).

It has been clearly established that structural changes in genomic stability in cancer are not fully selective, resulting in gain or loss in gene copy number. These changes in gene dosage perturb several genes that are co‐deleted or co‐amplified on large chromosome segments, known as “onco‐passenger genes.” A study by Mohanty and collaborators demonstrated that selective copy number methylation variation of these onco‐passenger genes is responsible for changes in their gene dosage. For example, collateral co‐amplification of genes in tumor suppressor pathways, such as TGF‐β and inflammatory signaling pathways, is compensated for by DNA hypermethylation to suppress their overexpression. On the contrary, the collateral deletion of pro‐oncogenic genes is compensated by DNA hypomethylation to promote their expression from the only remaining allele. In summary, these selective methylation mechanisms play a crucial role in regulating the expression levels of onco‐passenger genes, allowing an adaptive response to balance gene expression in the context of gene copy number changes in cancer.^101^

As evidenced by its evolutionary origins, regulatory circuits enabling gene dosage compensation can thus include both sensor systems at the protein level (change in the activity of a transcription factor) and effector systems at the transcriptional level. In mammals and fruit flies, the role of such noncoding RNAs (ncRNAs) when it comes to targeting genes affected by aneuploidy^24^, ^25^ has been examined. In particular, *Drosophila* has two ncRNAs on X (roX) targeting the MSL complex, which, as previously explained, is active only in male flies and is in charge of hyper‐activating the transcription of the single X chromosome.^22^ A human neuron mutagenesis study surprisingly revealed the roles of roX.^102^, ^103^ The mutational analyses of roX1 and roX2 in male flies showed that when mutations occur in both of them, the outcome is deadly for the fly, meanwhile one single mutation—in one or another roX—did not showed a deleterious effect nor any other type of differential phenotype.^104^ Evolution has aimed to secure the process of compensation, creating functional redundancy by first giving both roX different features, such as variation in size (3.7 kb vs. 0.5 to 1.4 kb), and creating a different binding site to MSL complex. This mechanism ensures the compensation process through the apparent sequence flexibility of the roX RNA. A conserved sequence known as the roX box is extremely important for the formation of conserved secondary structures and is critically present in multiple copies in each RNA.^21^, ^105^, ^106^ These structures help the protein complex to localize to the X chromosome and maintain active the acetylation of H4K16 allowing the transcription machinery to hyper‐function to account for dosage compensation.^22^

Different types of ncRNAs have been characterized and show a wide spectrum of functionality, mainly participating in the control of gene expression. In terms of genetic compensation, an RNA section of >200 bp could be predicted to function as a long noncoding RNA (lncRNA),^107^ interacting with transcription factors or modifying chromosomes to modulate regulatory regions of compensatory genes in homogeneous base pairs. Furthermore, other assays have also been carried out including the injection of short fragments of RNA (20–22 nt) giving an enhancement in transcription levels (Figure 2A). The method consisted in introducing RNA fragments into the cells, which would produce a double‐stranded RNA (dsRNA) with an antisense copy produced by the cell. This new formed dsRNA can use the RNA interference (RNAi) machinery to cause variations at chromosomal levels in an Argonaut (Ago)‐dependent manner, triggering addition of euchromatin histone tags or loss of heterochromatin histone tags. While the epigenetic mechanism underlying these mechanisms is not well elucidated, this hypothesis is coherent with other findings proving the transcriptional activation by modulating histones.^108^, ^109^ More studies have been performed in humans and mice, reinforcing the postulate of endogenous sequence segments that might participate in this anti‐transcriptional duplex dsRNA for compensation sites and finally transcriptional modulation.^110^

In order to cope with the disparity among female (XX) and male (XY) chromosomes, mammalian cells have adapted active mechanisms such as deactivation by the X‐inactivation gene (XIST), which in humans involves regulation through a lncRNA of 17 kb, with XIST been only active in the silenced—doubled—X chromosome^111^ throughout interphase.^112^ The inactivation process occurs during the initial stages of embryogenesis, during which it is possible to elucidate a number of variations in the doubled X chromosome suppressing transcription and overall genetic expression associated with X‐genes (explained as an intense “Barr body,” reviewed by Hall & Lawrence^113^). Nonetheless, there is also evidence of some genes “escaping” this inhibition^114^; however, autosomal chromatin has a substantial ability to be silenced.

In this same sense, XIST has been used to perform chromosomal silencing studies on chromosome 21 (Down syndrome), and it was found that overexpression of XIST leads not only to inactivation of the extra chromosome but also to a defined effect on the expression profile genomics that triggers stable heterochromatin modifications, chromosome‐wide transcriptional silencing, and DNA methylation to form a “chromosome 21 Barr body.” This initiates effects on heterochromatin, regulates transcription, and induces some biochemical variations such as DNA methylation. These findings hint at a possible therapeutic approach for patients with Down syndrome, as all of these hyperproliferative and neurogenetic effects might impact the brain.^115^

Among the vast number of possibilities utilized by cancer cells to cope with malignancy, they strive to make aneuploidy manageable. In 2021, Mohanty and collaborators conducted a multiomic study with The Cancer Genome Atlas (TCGA) including information of around 5.000 individual tumor samples to determine the coping strategies of cancer cells with high levels of aneuploidy. Using the criteria described above, they could observe bimodal distributions to classify all genes as coupled or uncoupled to their respective copy numbers. In cases of amplified genes, they found that coupled genes were related to cell cycle and EMT (pro‐tumor phenotypes), in contrast uncoupled genes that were related to patient cytotoxic immune infiltration and to a lesser extent with survival and apoptosis (anti‐tumor phenotypes). They could also observe a strong correlation between the degree of uncoupling and aneuploidy, indicating that large structural changes influencing the copy number of a substantial number of genes are associated with increased gene expression in coupled genes but not in uncoupled genes. This likely offsets any increase in the number of tumor‐toxic copy number changes and helps maintain tumor fitness. Authors emphasize how uncoupled mRNA expression from copy number for those genes plays a fundamental role in tumoral growth and maintenance, proposing these compensated genes as a therapeutic aim. In addition, they modeled gene expression for each gene expressed in cancer as a function of copy number, promoter methylation, regulating miRNAs and transcription factors with binding sites in the promoter and enhancers. The models were fitted using an elastic net and merged to construct cancer‐specific regulatory networks to compare the regulatory strength of the different factors. They found that gene uncoupling (compensation) is mediated by a complex orchestration of changes at the epigenetic (promoter methylation), transcriptional (transcription factors), and post‐transcriptional regulation (miRNAs). The dominant factor was the presence of transcription factors since more than 80% of uncoupled genes had at least one TF differentially expressed between uncoupled and coupled samples in a direction that explains uncoupling. Interestingly, they propose how these multiple mechanisms of compensation work together in tumoral cells resulting in cold tumors. Adding perturbation to their models in IRF1 and TRIM21, their overexpression caused a switch to a more pro‐inflammatory tumor type that could contribute with a better response to immune therapies in cancer.^72^

### 7.2 Post‐transcriptional control of gene expression at the RNA level

An additional possible mechanism that could trigger transcriptional adaptation has also been described that consists of RNA fragmentation as a triggering event. Mutations often produce messenger RNA (mRNA) with premature termination codons (PTCs), secondary structures that prevent ribosomal translocation or, less often, mRNA lacking stop codons. The presence of these mRNAs in turn leads to nonsense‐mediated decay (NMD) leading to degradation of the mRNA.^116^ In a study in zebrafish, it was shown that two mutations in the mt2 exon can cause different phenotypic intensities. Surprisingly, mutant alleles with a milder phenotype expressed higher levels of NMD. Antisense‐mediated reduction in the NMD pathway and decreased degradation of mutant mRNA resulted in a more severe phenotype, which is consistent with the ability of NMDs to induce a compensatory response to reduce the severity of the mutant phenotype.^117^

**FIGURE 3:**
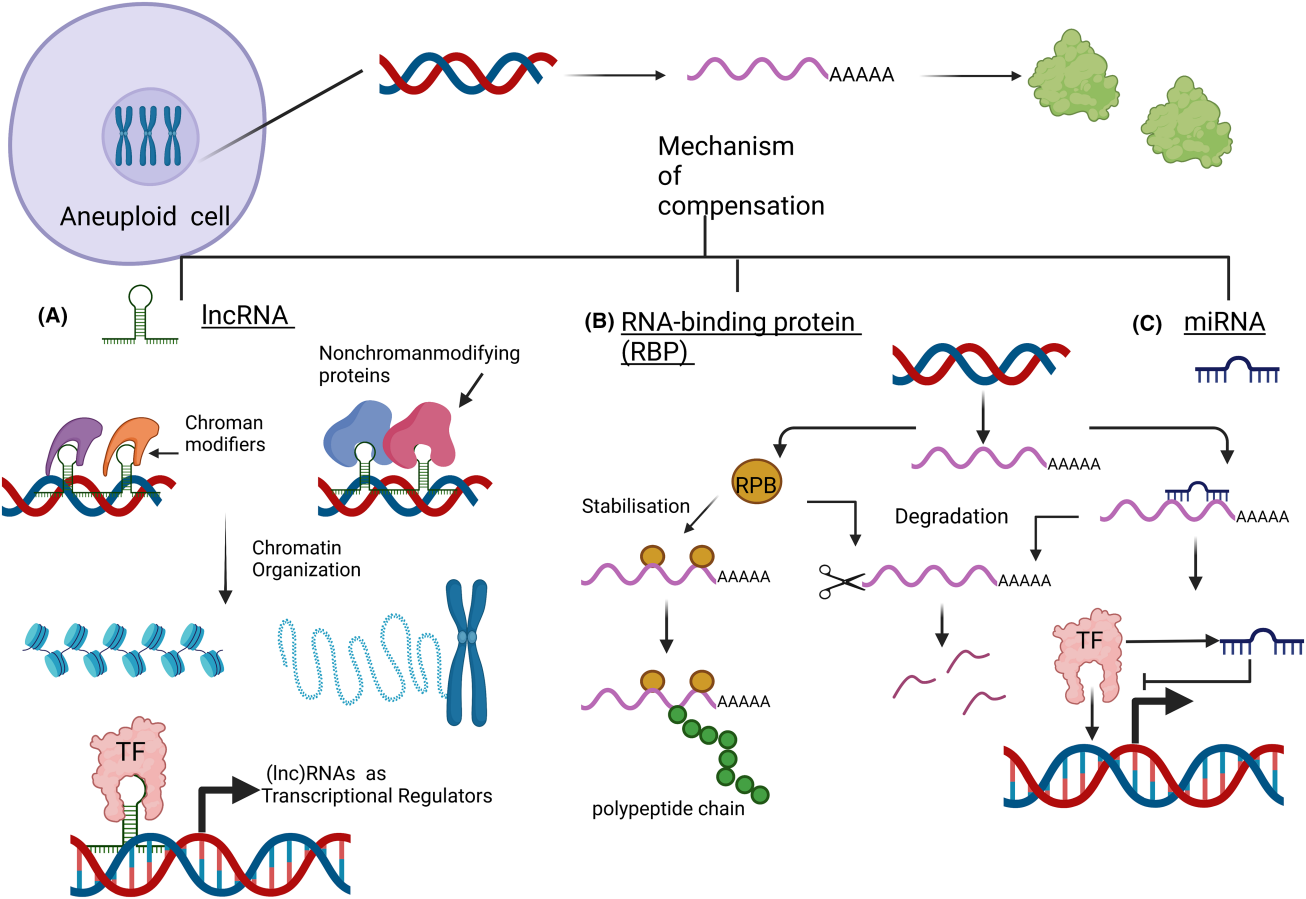
Mechanisms of gene dosage compensation at the transcriptional level. Regulation of gene expression by compensation through different mechanisms. (A) LncRNAs can modify gene expression by directing specific transcription factors or chromatin remodelers to the regulatory regions of compensated genes by homologous base pairing or by specifically regulating the binding of chromatin‐modifying or non‐modifying proteins. (B) RNA‐binding protein (RBP) can also regulate gene expression in several ways, one of which is to increase gene expression by stabilizing mRNA. (C) The miRNAs can regulate the expression of genes that have altered the number of copies, joining mRNAs by complementary homologous bases in the 3′UTR region or using complex networks that involve sensor loops between transcription factors (TF) and miRNA (figure generated using Biorender.com).

The RNA‐binding proteins (RBP) can also regulate gene expression in several ways, one of which is to increase gene expression by stabilizing mRNA (Figure 3B).^118^ The highly dynamic binding of RBPs is regulated by cellular conditions. For example, the regulatory mechanism of RBP interaction after genotoxic stress may be a mechanism by which cells compensate for gene loss. In this sense, proteins associated with mRNA coding function tend to be co‐regulated by specific RBPs, forming what is known as RNA operons or RNA regulators.^119^ Therefore, the same RBP could regulate compensated and mutant genes, and if the mutant mRNA is subject to degradation or if its secondary structure is affected by the mutation (thus affecting RBP binding), the RBPs would be available to stabilize the compensated genes.

One more potential mediator of gene dosage compensation are microRNAs (miRNAs) which play a pivotal role in routine regulation of transcription by using specific sequences to bind and either destroy or “block” the target mRNA and its pathway to protein.^120^ In recent years, miRNAs have been the group of noncoding RNAs found more frequently in clinical studies. miRNAs are endogenous molecules processed from kilobase‐long transcripts into mature sequences of ~22 bp in length, which recognize up to hundreds of specific targets through either perfect or imperfect complementarity. This characteristic of miRNAs is a particular feature that showcases their ability for high specificity but also redundancy on their targets and target pathways, counting around hundreds or thousands of genes that may fall under the regulatory function of a particular miRNA.^121^, ^122^

It is not fortuitous that miRNAs are a target molecules with therapeutic potential: within the human genome more than 2654 mature sequences for miRNA and almost 2000 hairpin precursors^123^ have been identified, together responsible for the regulation of approximately 60% of human genes.^124^ Hence, there is a good possibility that aneuploid cancer cells may be under the effect of dosage compensation mediated by a complex network of interactions between miRNAs and transcription factors (TFs) (Figure 3C).^71^

A well‐established function of miRNAs is gene silencing; however, they can also regulate gene expression through other mechanisms.^125^ It is clearly known that the target of miRNAs is mRNA, although it has been reported that miRNA‐373 can bind to CDH1 and CSDC2 promoter regions in PC3 cells (human prostate cancer cell line) and stimulates their expression by a mechanism that is not yet known (for review, see^96^). Likewise, miRNAs can increase the translation of some mRNAs; as when there is a deficiency of amino acids, miRNA10a binds to the 5′ UTR region of the ribosomal mRNA protein, improving its translation capacity and efficiency.^126^ It is also known that miRNAs have multiple mRNAs targets, so if there are mutations that lower the levels of mRNAs, the miRNAs already present in the cytoplasm target other mRNAs to regulate them.^127^

Moreover, miRNAs could regulate gene expression both at the post‐transcriptional RNA level and at the protein translational level, depending on their complementarity and binding to different argonaute (Ago) proteins.^105^, ^106^ This type of regulation could possibly fill the gap for those proteins regulated at the translational level and those compensated proteins that do not participate within heteromeric protein complexes. Taken together, all these mechanisms have a common organization principle, including a molecular mechanism to sense the change in copy number and an effector mechanism to regulate gene expression (Table 2).

**TABLE 2:** Mechanisms of gene dosage compensation including a dosage sensor and an effector on gene expression.

Species/condition	Dosage change	Sensor	Effector	References
Drosophila melanogaster	X chromosome in males	Male‐Specific Lethal (MSL) loci	Sequestration of general transcriptional regulators	[23]
Caenorhabditis elegans	Determination of phenotypic sex	Lethal Sex (Sxl)	RNA‐binding protein that regulates splicing and translation of key messenger RNAs in the sex determination and dose compensation pathways	[28]
Birds	Determination of sex	Doublesex and mab‐3‐related transcription factor 1 (DMRT1)	HEMGN, SOX9 y AMH Gonadal differentiation	[128]
Mammals	Compensation Chromosomes	MSL	roX (ncRNAs)	[22]
	Transcriptional regulation	Nonsense‐mediated mRNA decay (NMD)	Premature stop codons (PTC) RNA fragmentation	[116]
		lncRNA	Transcription factors or chromatin remodelers	[105, 106]
		iRNA	Chromatin modifications by dsRNA	[110]
		RNA‐binding protein (RBP)	Stabilizing mRNA RNA regulators	[119]
		Transcription factor activity	miRNAs	[71]
Cancer	Aneuploidy	Protein level	Excess subunits are degraded or aggregated	[86, 100]
		Cellular stress	eIF2α phosphorylation Increased translation of compensating genes	[95]
		Transcription factor activity	miRNAs	[71]
		Complex regulatory networks	Transcription factors, miRNAs, methylation	[72]

## 8 miRNAs AND GENE DOSAGE COMPENSATION MEDIATED BY SENSOR LOOPS

The work of 2021 of Mohanty and collaborators strongly indicated that transcription factor regulation plays a very important role in the regulation of gene expression uncoupling to copy number, followed by methylation and miRNAs.^72^ These individual elements can, however, interact and assemble regulatory networks. Indeed, transcription factors often participate in complex biological interactions. These biological interactions—natural and synthetic—must always show high levels of robustness in order to maintain homeostasis when facing fluctuations. Cells are constantly encountering variations, caused by changes in the efficiency of relative expression (and of course the availability of DNA template through epigenetics and/or mutations) but cells and their network plasticity are there to decouple the gene product when confronting alterations, ensuring whole network reliability. Biological networks often contain small‐scale subnetworks of recurring topologies called “motifs.”.^129^, ^130^

Indeed, miRNAs and transcription factors (TF) have been reported to couple and interact in the type of complex motifs forming endogenous transcription networks including, among them, positive, negative, coherent, and incoherent feedback loops. It should be noted miRNAs, TF, and target centers lead and influence nonlinear properties at the system level, such as oscillations, bistability, and ultrasensitivity.^130^, ^131^

Gene dosage compensation has been suggested to occur throughout all levels of the gene expression pathway—nonetheless, it is not well elucidated why or how the cell chooses to compensate at any given level. It has been found that certain regulatory points present some advantages. For example, when compensation occurs at mRNA levels using feedback switches, it might suggest a protection of mRNA levels upon chromosomal alterations (addition or deleting of regions), as has been found in fungi and yeast.^132^, ^133^ Mechanisms in mammals using incoherent feedforward motifs to regulate specific genes are also present, along with participation of miRNAs. Studies have uncovered loops including positive, negative, coherent, and incoherent feedbacks enriching the ability to modulate transitional or persistent patterns.^130^

**FIGURE 4:**
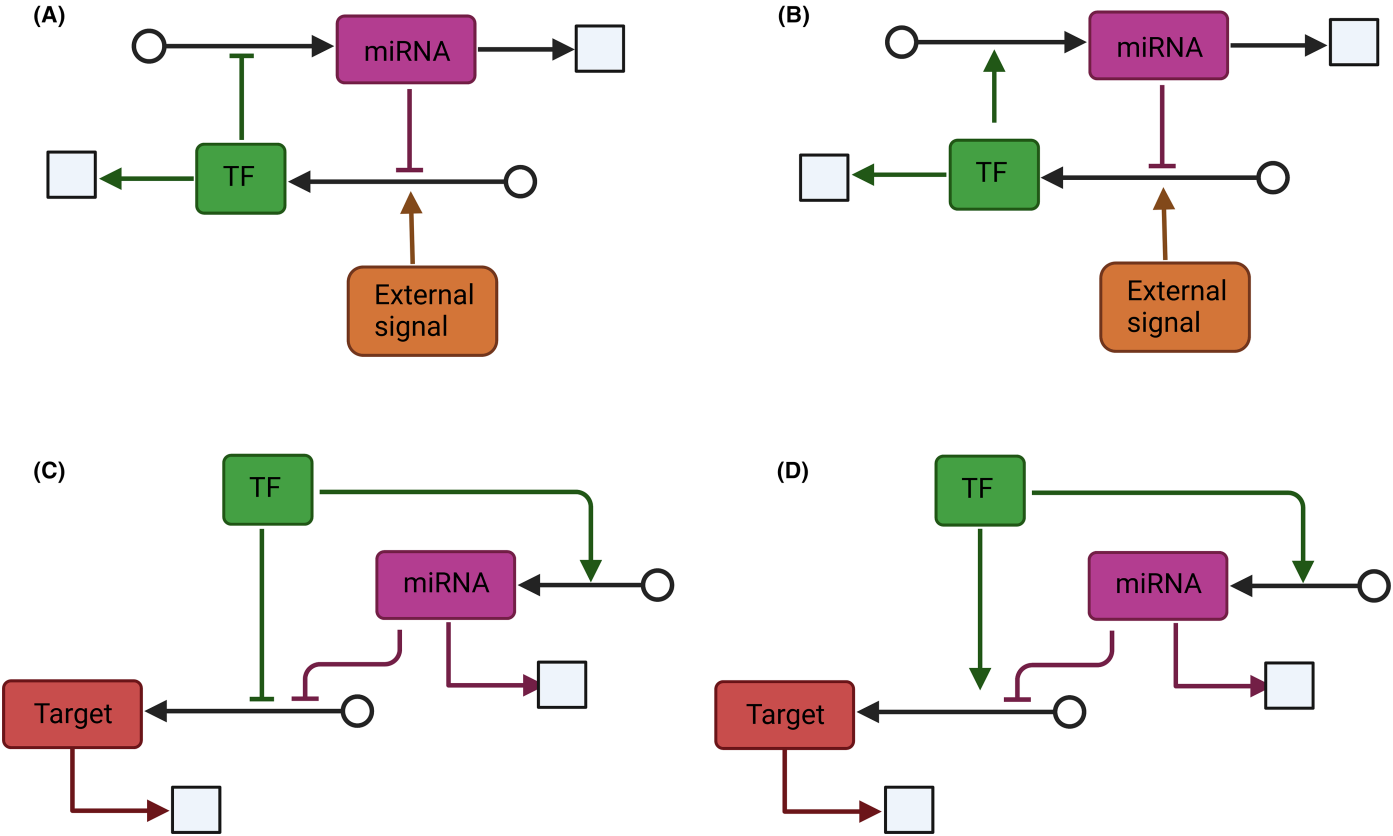
Diagram of motifs enables adaptive gene expression in mammalian cells. (A) Positive feedback loop mediated by miRNA. In positive feedback loops, transient external cues can lead to a permanent transition from a miRNA‐induced TF suppression scenario to a permanent and expressive TF‐mediated miRNA suppression scenario. (B) Negative feedback loop mediated by miRNA. During negative feedback loops, the system maintains homeostasis and increases external signals, and the enhancement of expression of TFs compensates by increased expression of miRNAs so that TF levels remain stable over a wide range of external signal values. (C) miRNA‐mediated coherent feedforward loop. Target expression by TFs is regulated in two ways: directly, by inhibiting transcription of the target gene, and indirectly, by enhancing the expression of miRNA that represses the target gene. (D) miRNA‐mediated incoherent feedforward loop. Target expression by TFs is regulated in two ways: positively, by direct activation of the expression of target genes, and negatively, by enhancing the expression of miRNAs that repress target genes (figure generated using Biorender.com).

The dynamics between miRNAs and other several molecular contributors demonstrate the intricate interplay between the various mediators involved. As an example, we have the well‐studied protein p53, which presents a complicated network with a negative regulator NAD‐dependent deacetylase sirtuin‐1 (Sirt1) and its repressor mir‐34a (Sirt1⊣p53 → miR‐34a⊣Sirt1; see^131^). Although feedback loops, specifically the positive ones, are meant to create a homeostatic expression including both miRNAs and TF, exogenous disruptions can lead to irreversible states of miRNA silencing, inducing an overexpression of TFs (Figure 4A). This means that positive feedback loops are susceptible to transforming a transient signal into a long state cellular response,^134^ perhaps creating more harmful consequences such as signal amplification and instability.^135^

Negative feedback loops are formed when a molecule is in charge of producing its own inhibitor. In negative feedback loops involving miRNAs, TFs produce miRNAs to modulate themselves (Figure 4B). This type of loop is constantly leading to termination or regulation by fluctuation of its components, being part of the simplest systems for balance maintenance or robustness—by responding to gene necessities only when required while there are non‐controlled variables changing in the surrounding.^136^

Coherent feedback loops offer gene regulation by immediate TF‐related networks, with coherent direct loops where genes are activated directly by TFs and directly via miRNA inhibition (double positive) and loops that repress genes directly through TFs binding or through repression of the target gene indirectly by miRNA activation (double negative regulation) (Figure 4C). When miRNAs are involved in these types of loops, they generally act by delaying the gene response, being called signal‐sensitive delay factors.^136^

Moreover, the incoherent type of loop is oppositely regulated using both TF and miRNA (Figure 4D). These loops are divided into two types: type A, in which the target gene suffers from activation by TFs and the indirect effect of miRNAs; type B, in which genes are repressed by TFs and through miRNAs indirectly activated by TFs.^130^ As a result, it has been possible to elucidate the availability of DNA template in in silico projects and regulate the gene expression, supporting a previously putative endogenous role in gene dosage compensation for such motifs.^129^, ^137^

Genetic dose compensation has been widely investigated, elucidating how very complex networks of miRNAs and TFs work together as a natural homeostatic mechanism. Also elucidated is the process by which tumor cells have adapted this system to withstand the negative consequences of proteotoxicity given aberrations in cancer karyotypes. By using computational models, the data from NCI‐60 cancer cell lines were analyzed to propose how these networks perform dosage compensation at the transcriptional level and hence identify genes with really short ranges of fluctuation at the mRNA and protein levels despite enormous alterations in copy numbers, as described in the section referring to the criteria to identify gene dosage compensation.^71^ We found that Myc can be designated as one of the genes with the highest copy number variation (CNV) but lowest variation in expression, fitting our criteria for a gene dosage‐compensated candidate.^71^

Our research team was able to identify a very complex network of interactions between TFs, miRNAs, and the compensated genes. Using Systems Biology, a mathematical model was constructed to include dynamic models and all the genomic copy number, transcriptional expression, and proteomic data available from the NCI60 panel. The model includes what is called “ordinary differential equations” (ODE) that use mass action kinetics to finally show all the dynamics between the elements previously mentioned. Using sensitivity analyses and biological simulations, it was possible to identify what we called a minimal model of dosage compensation for the oncogene Myc extracting the most important properties of the enormous initial network of interactions to determine that *Myc* forms three feedback loops with the miRNAs: miR17, miR19a, and miR20a, each of them redundantly compensating the expression of this gene upon copy number amplifications. In addition, the model parameters were mapped in a region within the multidimensional parameter space enabling dosage compensation. Therefore, it is crucial to emphasize that the network topology by itself is not sufficient, but lends a perfect balance of the strengths of those interactions within a range of gene dosage compensation. In addition, we also demonstrated that Myc‐amplified cell lines were more susceptible to the inhibition of gene dosage compensation.^71^

We also confirmed our findings with CCLE and TCGA datasets, indicating that Myc dosage compensation is active in most tumor types, despite the high degree of heterogeneity observed in cancer.^71^ Our results are consistent with the description provided by Zhao et al, who analyzed gene expression in GliNS2 cancer stem cells (CSCs) and CB660 neural stem cells (NSCs). They found that the transcription factor c‐MYC is a single gene whose expression shows differential transcription number‐dependent gene expression.^138^ Other reports cited in this review also found Myc as one of the most compensated genes with all their corresponding criteria to identify gene dosage‐compensated genes.^43^, ^70^, ^73^ As stated above, all these combined criteria can help to identify candidate genes but a full definition of a functional gene dosage compensation requires further experimental validation (see below).

In conclusion, the sensor loops or regulatory circuits described enabling gene dosage compensation fit the general framework of dosage compensation systems, including a sensor (change in the activity of a transcription factor) and effector (a miRNA to suppress gene expression), a property that directly depends on the parameter values governing these interactions. We need to point out that this mechanism could account for gene dosage compensation both at the RNA and protein levels possibly depending on the miRNA‐mRNA type of interaction and the type of argonaute protein involved.

## 9 EXPERIMENTAL DEMONSTRATION OF GENE DOSAGE COMPENSATION

As discussed above, chromosomal variations are constantly happening through sex‐related phenomena and mutational events, and hence, evolution leads cells to cope with this burden. Nevertheless, scientists have not yet determined whether there is an upper limit of copies with which a cell can cope. Ishikawa et al. used modified yeast strains to express a protein with a tandem affinity purification (TAP) tag in an endogenous gene, which could be easily detected by fluorescence. Additionally, they introduced a multi‐copy plasmid containing the same protein but with no tag (exogenous gene). In the absence of compensation, a proportional increase in the exogenous protein expression was expected without a change in the expression of the endogenous protein. However, in the presence of gene dosage compensation, the endogenous protein showed an unexpected behavior by decreasing its quantity in the presence of the exogenous version. Investigators suggested the discovery of dosage compensation at protein level and a methodological approach to quantify it.^84^

Given the nature of the experiments and the behavior established among protein overproduction and compensation mechanisms, the name “*tug‐of‐war*” was proposed for the complex interplay between aneuploidy and compensation.^84^ Our research team saw potential in using these principles to develop a similar technique to advance from the minimal model of compensation in silico to the experimental validation for the compensation circuits of *Myc*, a gene that is overexpressed in ~60% of cancers, and the validation of *Myc‐*associated dosage compensation as a possible target of cancer cell therapy.^71^

Similar principles were taken from the protein level “*tug‐of‐war*,” but in our case we aimed to demonstrate gene dosage compensation at the transcriptional level, driven by miRNAs. miRNAs dock with a protein complex and bind with either total or partial complementarity primarily to the 3′UTR segment of target mRNAs, leading to either mRNA cleavage (transcriptional level) or repression of translation (protein level). For this work, we focused on transcriptional regulation leading to a downregulation of the expression of the target mRNA. We designed a plasmid containing a functional copy of *Myc* but lacking the 3´UTR fragment (exogenous gene), and transfected it into cancer cell lines already having *Myc* amplifications in order to exert additional pressure on those compensating feedback loops. We used differential codon optimization while constructing the exogenous version of *Myc* and standardized a specialized RT‐PCR protocol allowing us to distinguish endogenous *Myc* from the exogenous version. As expected, the overexpression of exogenous *Myc* at 24 h and 48 h leads to a concomitant downregulation of endogenous *Myc*. The sharp increase in the exogenous gene expression strongly suggests that the compensation occurs by the proposed mechanism mediated by miRNAs at the level of transcription, forcing the downregulation of the only available target: endogenous *Myc*.^71^

Moreover, we evaluated the effect of interrupting the minimal model of compensation proposed for *Myc* through the use of specific anti‐miRs which acts as “decoy targets” for miR17, miR19a, and miR20a, thus interfering with compensation. To evaluate this protein‐dependent‐toxicity, three colon cancer cell lines were used, including *Myc* copy numbers of 2, 4, and 7. We could observe a correlation between copy number and sensitivity to the inhibition of the respective mechanism, suggesting that Myc was already set to an upper limit of expression to support malignancy, related to aneuploidy that the cell can handle. Finally, we fitted individual models of TCGA patient data with breast cancer and could observe an inverse relationship between patient survival and Myc dosage compensation. This experimental demonstration completes a full definition of a functional gene dosage compensation for the Myc oncogene.

Taken together, the techniques shown here for the experimental validation of gene dosage compensation both at the transcriptional and protein levels could be combined with cytotoxicity studies to confirm a functional compensation with therapeutic potential against aneuploid cancer.

## 10 CONCLUSIONS

Given that a specific chromosome fragment can contain tens to hundreds of genes, even small alterations in the structure or the number of chromosomes can cause deleterious effects. Indeed, the larger the affected chromosomal region, the stronger the lethal outcome for the cell. Embryonic alterations at chromosomal levels (not including sex‐related ones) are known to cause death, and the few ones who make it through adulthood carry severe consequences. However, recent studies of dosage compensation in cancer have made it abundantly clear that some cells modulate regular homeostatic pathways and use them to reduce the negative impacts of aneuploidy on their path to becoming a fully developed and well‐established cancer.

Regulatory cell pathways are neither linear nor simple; they work as complex networks involving regulatory motifs such as positive, negative, coherent, and incoherent loops that work either forward or in a feedback mechanism to control the gene expression. One pivotal pathway when overcoming aneuploidy is gene dosage compensation, a mechanism with robustness sufficient to sustain stable protein levels even when there is an abnormal number of gene copies, hence downregulating or upregulating its expression to meet the cell necessities.

Of the hundreds to thousands of genes dysregulated by aneuploidy, there are some that require the compensation mechanism for their precise function. Therefore, the most critical question is whether it will be possible to identify when and how a specific compensated gene can be targeted to block dosage compensation and induce an error catastrophe in cancer. Systems Biology and in silico technologies come to “simplify” the identification of the complex networks regulating this stability, and ncRNAs appear to be a critical and integrated part of not only complex network regulation but of the potential therapeutic approaches currently under study.

The criteria reviewed here to identify gene dosage compensation are diverse but the right combination could be used to narrow down the list of compensated candidates for further experimental validation using genetic tug‐of‐war approaches both at the mRNA and protein levels to confirm the presence of compensation circuits that could be specifically targeted. These approaches combined with cytotoxicity experiments could enable the identification of functional gene dosage compensation and the specific mechanisms to be targeted in highly specific therapeutic interventions against aneuploid cancer.

Current research efforts aim to fully understand the network of compensated genes in cancer in order to identify targets enabling cancer cell robustness to the genome‐wide copy number alterations observed in aneuploidy. The in silico models and the experimental platforms developed with such purpose represent an initial approach for the screening of potential therapeutic molecules aiming to modulate such key targets for aneuploid cancer robustness.

## AUTHOR CONTRIBUTIONS

**Diana Bravo‐Estupiñan:** Writing – original draft (equal); writing – review and editing (equal). **Karol Aguilar‐Guerrero:** Writing – review and editing (supporting). **Steve Quirós:** Supervision (equal); visualization (equal). **Man‐Sai Acón:** Visualization (equal); writing – review and editing (equal). **Christian Marín‐Müller:** Writing – review and editing (supporting). **Miguel Ibáñez‐Hernández:** Writing – review and editing (equal). **Rodrigo Mora‐Rodríguez:** Supervision (equal); visualization (equal); writing – review and editing (supporting).

## FUNDING INFORMATION

This work was supported by UCREA, Vicerrectoría de Investigación, University of Costa Rica, Grant C1468. R.M. has received research support from the Alexander von Humboldt Foundation, Germany.

## CONFLICT OF INTEREST STATEMENT

The authors have no relevant financial or non‐financial interests to disclose.

## ETHICS STATEMENT

This work does not involve human subjects.

## CONSENT TO PARTICIPATE

This work does not involve human subjects.

## CONSENT TO PUBLISH

This work does not involve human subjects.

## Data Availability

No new data were created or analyzed in this study. Data sharing is not applicable to this article.
